# Comprehensive Analysis of CircRNA Expression Profiles in Multiple Tissues of Pigs

**DOI:** 10.3390/ijms242216205

**Published:** 2023-11-11

**Authors:** Qingpeng Shen, Wentao Gong, Xiangchun Pan, Jiali Cai, Yao Jiang, Mingran He, Shanghui Zhao, Yipeng Li, Xiaolong Yuan, Jiaqi Li

**Affiliations:** 1 Guangdong Laboratory of Lingnan Modern Agriculture, National Engineering Research Center for Breeding Swine Industry, State Key Laboratory of Swine and Poultry Breeding Industry, Guangdong Provincial Key Laboratory of Agro-Animal Genomics and Molecular Breeding, College of Animal Science, South China Agricultural University, Guangzhou 510642, China; qingpeng_shen@stu.scau.edu.cn (Q.S.); g_w_tao@163.com (W.G.); pxc_816@126.com (X.P.); jlcai_scau@163.com (J.C.); jyao192@163.com (Y.J.); mingranhe@stu.scau.edu.cn (M.H.); pagexzsh@163.com (S.Z.); hi0530_liyipeng@126.com (Y.L.); 2 School of Medical, Molecular and Forensic Sciences, Murdoch University, Murdoch, WA 6149, Australia

**Keywords:** pigs, housekeeping circRNAs, tissue-specific circRNAs

## Abstract

Circular RNAs (circRNAs) are a class of non-coding RNAs with diverse functions, and previous studies have reported that circRNAs are involved in the growth and development of pigs. However, studies about porcine circRNAs over the past few years have focused on a limited number of tissues. Based on 215 publicly available RNA sequencing (RNA-seq) samples, we conducted a comprehensive analysis of circRNAs in nine pig tissues, namely, the gallbladder, heart, liver, longissimus dorsi, lung, ovary, pituitary, skeletal muscle, and spleen. Here, we identified a total of 82,528 circRNAs and discovered 3818 novel circRNAs that were not reported in the CircAtlas database. Moreover, we obtained 492 housekeeping circRNAs and 3489 tissue-specific circRNAs. The housekeeping circRNAs were enriched in signaling pathways regulating basic biological tissue activities, such as chromatin remodeling, nuclear-transcribed mRNA catabolic process, and protein methylation. The tissue-specific circRNAs were enriched in signaling pathways related to tissue-specific functions, such as muscle system process in skeletal muscle, cilium organization in pituitary, and cortical cytoskeleton in ovary. Through weighted gene co-expression network analysis, we identified 14 modules comprising 1377 hub circRNAs. Additionally, we explored circRNA–miRNA–mRNA networks to elucidate the interaction relationships between tissue-specific circRNAs and tissue-specific genes. Furthermore, our conservation analysis revealed that 19.29% of circRNAs in pigs shared homologous positions with their counterparts in humans. In summary, this extensive profiling of housekeeping, tissue-specific, and co-expressed circRNAs provides valuable insights into understanding the molecular mechanisms of pig transcriptional expression, ultimately deepening our understanding of genetic and biological processes.

## 1. Introduction

Circular RNA (circRNA) is a type of non-coding RNA generated through a unique splicing process known as backsplicing [1]. Backsplicing is a specific mechanism that produces downstream splice-donor sites and upstream splice-acceptor sites, resulting in their covalent connection [2]. Various types of circRNAs have been identified, including exon, intron, and exon–intron type [3]. Most circRNAs (exonic circRNAs) are expressed from known protein-coding genes and consist of a single exon or of multiple exons [4,5]. Additionally, a minority of circRNAs (intronic circRNAs) are composed of introns [6]. Other circRNAs (exon–intron circRNAs) contain sequences derived from both exons and introns due to internal intron retention or a failure in the debranching of intronic lariats during canonical splicing [7,8,9]. The circular structure of circRNA gives it higher stability and resistance to degradation. This particular structure of circRNA allows circRNAs to persist and remain stable in cells for extended periods of time [10]. Meanwhile, circRNAs have a range of biological functions, including competitive miRNA inhibition, direct regulation of parental mRNA expression, and protein sequestration [11,12,13].

Pigs exhibit a significant degree of physiology and anatomy similarity to humans, rendering them a favorable animal model for investigating human diseases and developmental processes [14]. Moreover, domestic pigs are essential farm animals with a long history of selective breeding and immense economic value [15]. Over time, humans have conducted extensive breeding programs for domestic pigs, resulting in a rich genetic diversity and the development of various desirable traits [16]. Research on circRNAs is an important area of study for pig breeding, as differential expression of circRNAs has been shown to impact various traits, including meat quality, reproductive ability, and disease susceptibility. For instance, Li et al. found that circIGF1R could act as a miRNA sponge to negatively regulate miR-16 to promote the myoblast differentiation of the porcine skeletal muscle satellite cells, thereby influencing meat quality [17]. Wang et al. detected 60, 78, and 86 differentially expressed circRNAs that played a key role in muscle development and lipid deposition across 38, 58, and 78 days post conception [18]. Zhuang et al. identified circKANSL1L, which influences muscle fiber type differentiation and exhibits a high level of conservation between mice and pigs [19]. Niu et al. detected 305 differentially expressed circRNA in porcine ovaries, and they were enriched in several reproductive-related signaling pathways [20]. Mester-Tonczar et al. demonstrated that circRNA-CDR1as beneficially impacts cardiac function in pigs by down-regulating miR-7 in the heart [21]. Although there have been some advances in pig circRNA research over the past few years, investigations are still mainly focused on a limited number of tissues.

The effect of circRNA on pig traits constitutes a complex regulatory network involving multiple processes and mechanisms. A better comprehension of the circRNA profiles in different pig tissues will help us to leverage the pig genome for applications in various fields, such as species of evolution, growth and development, breeding, construction of models for humans, and gene–disease phenotypic association analysis. With the advancement of RNA sequencing technology, an increasing number of pig genomic data have been made publicly available and widely utilized [22]. Numerous studies of non-coding RNA based on RNA-seq and the related databases have been published [23,24]. The databases of circRNA were published as well, such as CircFunBase and CircAtlas [25,26], but the number of circRNAs in pigs should be greatly enriched. Furthermore, recent studies have found a significant amount of circRNA in porcine muti-tissues. For example, Jin et al. found 48,232 circRNAs from 31 tissues and two cell lines [27]. However, circRNA expression in multiple tissues of pigs should still be explored based on bulk data.

In this study, we aimed to investigate the circRNA profiles by RNA-seq from nine porcine tissues (gallbladder, heart, liver, longissimus dorsi, lung, ovary, pituitary, skeletal muscle, and spleen) to explore the association and distinctness of circRNA expression patterns among different tissues. Our observations will provide new insights into the biological processes of various tissues in pigs.

## 2. Results

### 2.1. CircRNA Detection in Multiple Tissues

**Figure 1:**
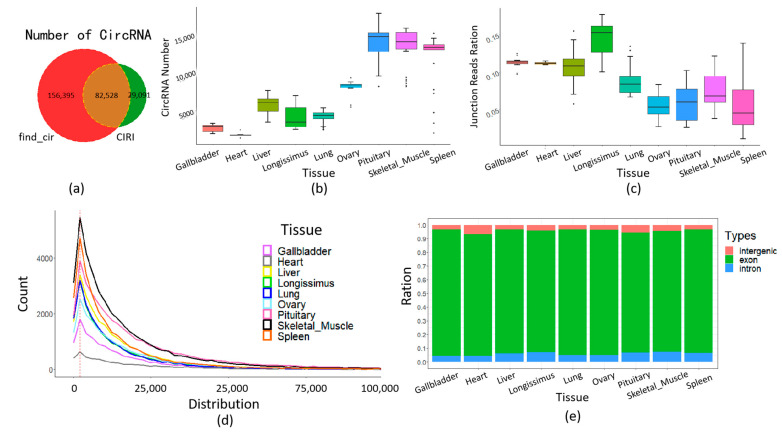
Descriptive statistics of the identified circRNA. (**a**) Number of circRNA detected by find_cir and CIRI. (**b**) CircRNA numbers among different tissues. (**c**) CircRNA junction ratio among tissues. (**d**) CircRNA length distribution among tissues. (**e**) CircRNA types among tissues.

CircRNAs were detected using the CIRI2 and find_cir methods in a total of 215 samples from nine different tissues (see Section 2 for details). In summary, 238,923 circRNAs were detected using the find_cir, while 111,619 circRNAs were identified utilizing the CIRI2. Among these, a total of 82,528 circRNAs were detected by both methods and used for subsequent analysis (Figure 1a, Table S2). Compared with the CircAtlas database (https://ngdc.cncb.ac.cn/circatlas/, accessed on 7 November 2023) [26], in total, 3818 new circRNA were found (Table S3). The tissue-specific distribution of circRNAs indicated that the highest number (14,224) of circRNAs was detected in the pituitary, while the lowest number (1709) was detected in the heart compared to other tissues (Figure 1b). Additionally, the longissimus dorsi exhibited the highest mean junction ratio (0.15) among the tissues, while the spleen showed the lowest mean junction ratio (0.06) compared to other tissues (Figure 1c). The average genome distance of all circRNAs was calculated to be 15,383 bp, with circRNAs shorter than 50,000 bp accounting for 93.9% of the total (Figure 1d). Notably, the majority of circRNAs belonged to the exonic type, accounting for 88.0% (pituitary) to 92.6% (gallbladder) across different tissues. The remaining circRNA types, including intronic and intergenic, accounted for 4.2% (gallbladder) to 7.4% (skeletal muscle) and 3.2% (gallbladder) to 6.8% (heart), respectively (Figure 1e).

### 2.2. CircRNA Clustering Analysis

**Figure 2:**
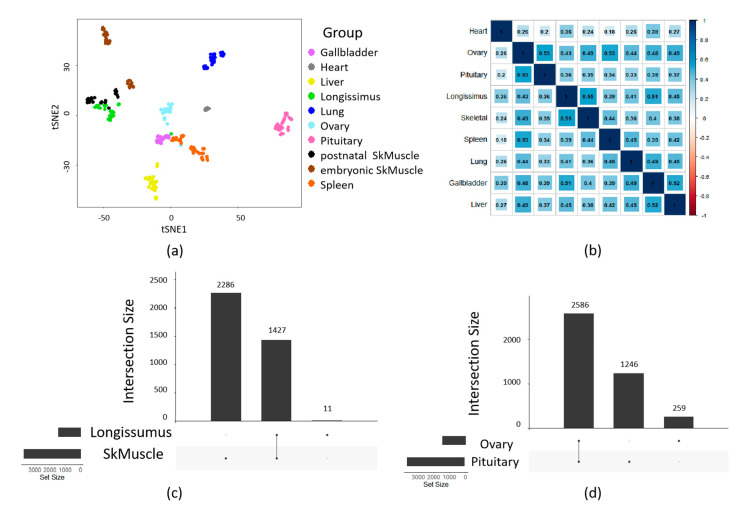
CircRNA expression among tissues. (**a**) t−SNE analysis of tissues. (**b**) Spearman’s correlation among tissues. (**c**) CircRNA parental gene between ovary and pituitary. (**d**) CircRNA parental gene between longissimus dorsi and skeletal muscle.

Clustering analysis was performed on the retained circRNAs, and the clustering results demonstrated that most tissues exhibited well-defined clusters. However, a noticeable stratification was observed within the skeletal muscle tissues (Figure 2a). Principal component analysis (PCA) showed that the skeletal muscle samples could be classified into two distinct groups based on their developmental stages: embryonic muscle and postnatal muscle (Figure S1).

Furthermore, circRNA average expression levels across tissues were used to perform Spearman clustering analysis. Specifically, a strong correlation was observed between skeletal muscle and the longissimus dorsi (*r* = 0.55), indicating a similarity in the expression profiles of circRNAs in these tissues. Similarly, a significant correlation was found between the ovary and pituitary gland (*r* = 0.53) (Figure 2b).

Through an analysis of the parental genes associated with circRNAs between tissues, it was observed that more than 99% (1427/1438) of circRNAs in the longissimus dorsi tissue were shared with skeletal muscle (Figure 2c). On the other hand, circRNAs shared by the pituitary gland and ovary exhibited a high degree of overlap in their parental genes, with over 50% (2586/4091) of these host genes being shared with both tissues (Figure 2d).

### 2.3. Tissue-Specific and Housekeeping CircRNA Profile

**Figure 3:**
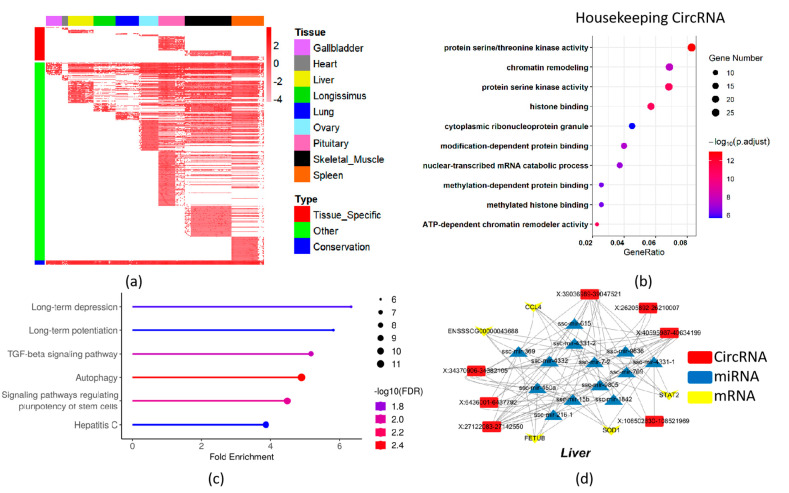
CircRNA expression profile and function prediction. (**a**) CircRNA profiles in all samples. (**b**) GO enrichment of housekeeping circRNAs. (**c**) KEGG enrichment of housekeeping circRNAs. (**d**) ceRNA network of liver.

After a strict screening (see Section 2 for detail), in total, 24,745 circRNAs were identified by pervious materials in this study, of which 492 were categorized as housekeeping circRNAs and 3489 were classified as tissue-specific circRNAs (Figure 3a, Table S4). Furthermore, 403 of the 492 housekeeping circRNAs’ parental genes were housekeeping genes identified by the pigGtex project (Table S5). To gain insights into the biological functions of these circRNAs, the parental genes (housekeeping circRNAs) associated with them were subjected to GO pathway analyses. The GO enrichment analysis of housekeeping circRNAs enriched in chromatin remodeling and nuclear-transcribed mRNA catabolic processes in the biological process category, like histone binding (Figure 3b, Table S6), suggested that housekeeping circRNAs may be involved in gene expression regulation and RNA metabolism. In the tissue-specific circRNAs, the parental genes are also able to be enriched for their associated functions, for instance, muscle system process in skeletal muscle and neuron-to-neuron synapse in pituitary (Figure S2, Table S7).

We found that the tissue-specific circRNAs were significantly related to traits of meat and carcass, and health and reproduction (Table S10). Additionally, the KEGG pathway analysis of housekeeping circRNAs revealed significant enrichment, such as long-term depression, long-term potentiation, TGF-beta signaling pathway, and autophagy (Figure 3c, Table S8). These pathways are known to play crucial roles in various cellular processes and signaling cascades. Similarly, the parent genes of tissue-specific circRNA are also able to be enriched for their associated functions (Figure S3).

Furthermore, the miRNAs with the top five highest scores in miRanda-based circRNAs match were selected as potential miRNA targets (see Section 2 for detail). We found that tissue-specific circRNA might interact with many miRNAs or indirectly with differentially expressed tissue-specific genes in liver, longissimus dorsi, lung, ovary, and pituitary tissues (Figure S4). For instance, we found that “circ X:27122083-27142550” might interact with STAT2 via ssc-mir-615, might interact with CCL4 via ssc-mir-1842, and might interact with FETUB via ssc-mir-7-2 in liver (Figure 3d).

### 2.4. Co-Expression CircRNA Network across Pig Tissues

**Figure 4:**
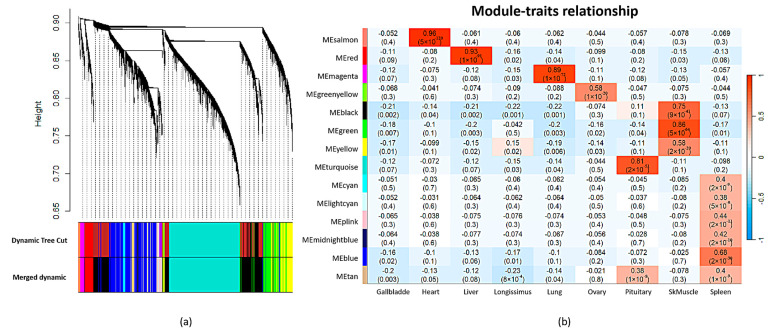
WGCNA of circRNAs. (**a**) Cluster of circRNAs. (**b**) Relationship between module and tissues.

WGCNA was performed on the filtered circRNAs to investigate the biological relationships and potential functions of the core circRNAs among tissues. In this analysis, a soft threshold value (*β*) was determined to construct an efficient scale-free network. When the R^2^ (coefficient of determination) value exceeded 0.8, the threshold value of 4 was selected (Figure S5). By applying the hierarchical clustering algorithm, in total, 16 distinct modules were obtained. These modules were subsequently corrected and merged, resulting in 14 modules (Figure 4a and Figure S5). Each module represents a set of circRNAs that exhibit highly correlated expression patterns across tissues. Among these modules, seven module–tissue relationships were tissue-specific modules (*r* > 0.65). For instance, the salmon module displayed a strong correlation with heart (*r* = 0.96), and the red module showed a strong correlation with liver (*r* = 0.93), and the magenta exhibited a strong correlation with lung (*r* = 0.89) (Figure 4b).

Furthermore, among these seven tissue-specific modules, we applied the MM and GS methods to identify hub-circRNA genes by using a cutoff of |GS| > 0.2 and |MM| > 0.8. Finally, 142, 262, 160, 149, 29, 625, and 10 hub circRNAs were observed in heart—salmon, liver—red, lung—magenta, skeletal muscle—black, skeletal muscle—green, pituitary—turquoise, and spleen—blue modules, respectively (Table S9).

### 2.5. Conservation between Pig and Human

We compared the circRNAs between the pig and human genomes using the LiftOver tool (minMatch = 0.5). We found that 77.10% of circRNAs (19,078/24,745) in the pig genome could be aligned with sequences in the human genome. Furthermore, 19.29% of the pig circRNAs (4774/24,745) were sequenced and usage conserved with circRNAs in the human genome (Table 1). These conserved circRNAs are all derived from orthologous genes between pig and human (Table S11), and 96% of the conserved circRNA originate from exons (Figure S6).

## 3. Discussion

Pigs serve as an essential economic resource and an ideal animal model for studying various aspects of animal domestication and human diseases. In recent years, circRNAs have emerged as a prevalent class of non-coding RNAs with diverse functions. However, the pattern of circRNA expression based on large-scale data from diverse tissues remains unclear in pigs. In our study, we aimed to comprehensively explore the landscape of circRNAs in pigs by analyzing a dataset comprising nine different pig tissues. In total, 3489 tissue-specific circRNAs and 492 housekeeping circRNAs were preliminarily identified. Meanwhile, 14 modules with 1377 hub circRNAs were obtained.

To ensure the accuracy of the results, we employed two well-established algorithms, CIRI2 and Find_cir, to detect circRNAs using RNA sequencing data. CIRI2 algorithm leverages paired chiastic clipping signals obtained from the mapping information, while Find_cir predicts back-splicing events by examining the first and last 20 bp anchors of unmapped reads [28,29]. These algorithms have been extensively validated and used in previous studies [30]. It is important to note that due to differences in their underlying principles and strategies, these algorithms may yield different predictions for circRNAs. Hence, we adopted a conservative approach by combining the outputs of both algorithms to minimize false-positive predictions and enhance the reliability of our circRNA dataset. By this approach, more than 95% of detected circRNA could be found in the released database which could certify the accuracy of this method [31].

Statistical analysis of the detected circRNAs revealed that exon-derived circRNAs accounted for approximately 90% of the total circRNAs across pig tissues. This observation aligns with previous studies in pigs and other species [30,32]. Additionally, we observed that the distribution of circRNAs peaked within a genomic span of 2000 bp, with a gradual decrease in abundance as the genomic span increased. This distribution pattern is consistent with known characteristics of circRNAs [30,33]. Furthermore, our study revealed tissue-specific differences in the quantity and splicing rates of circRNAs. This suggests that circRNA biogenesis and regulation are subject to tissue-specific factors, such as alternative splicing and post-transcriptional processing. These tissue-specific differences highlight the potential functional diversity and regulatory roles of circRNAs in different biological contexts [34,35].

The expression analysis of circRNAs in pigs revealed distinct tissue-specific expression profiles across different tissues. Subsequently, the study further investigated and identified 3489 tissue-specific circRNAs and 492 housekeeping circRNAs. By performing pathway enrichment analysis on the parental genes of tissue-specific circRNAs, key pathways related to tissue-specific functions were uncovered. Taking the heart tissue as an example, several parental genes were found to be involved in the formation of intercellular junction complexes. For instance, the parental genes PKP2 of “circ5:41492479|41493703” and “circ5:41438437|41447707” and CDH2 of “circ6:112408292|112462754” interact with other proteins, such as intercellular adhesion proteins and cytoskeletal proteins, enhancing the adhesion and stability between cardiac muscle cells [36,37]. In the liver, the parental gene GYS2 of “circ5:51884381|51889554” plays a role in glycogen synthesis and metabolic regulation [38]. In the longissimus dorsi, the parental gene CACNA1S of “circ10:23546471|23549237” encodes the α1S subunit of a voltage-gated calcium ion channel, which is predominantly expressed in skeletal muscle cells and regulates muscle contraction [39]. In the lungs, the parental gene CTSS of “circ4:98422323|98425826” is associated with airway inflammation and lung disease development, including its involvement in lung cancer progression [40]. In the ovaries, the parental gene PBX1 of “circ4:85892566|85906932” plays a critical role in embryonic development, contributing to processes such as axon guidance, organ formation, and segmental differentiation [41]. In the pituitary gland, the parental gene ADCY9 of “circ3:38275930|38288061” encodes adenylate cyclase 9, which catalyzes the cyclization of adenosine monophosphate and generates the second messenger cyclic adenosine monophosphate (cAMP) [42]. In skeletal muscle, the parental gene ACTN2 of “circ14:54704317|54716478” encodes alpha-actinin-2, a myofibrillar protein responsible for constructing and maintaining the cell cytoskeleton. ACTN2 plays a crucial role in muscle contraction and force transmission [43]. In the spleen, the parental gene ITGAL of “circ3:17840569|17841414” regulates the activity and function of immune cells by participating in their adhesion, migration, and interactions within the spleen [44]. Notably, specific circRNAs in the gallbladder tissue, such as SLC4A4 and SLC5A1, are primarily involved in gastrointestinal tract-related functions [45,46]. These findings highlight the functional diversity and tissue-specific roles of circRNAs in different organs. The identified parental genes associated with tissue-specific circRNAs provide insights into the potential regulatory mechanisms and biological functions of circRNAs in specific tissues.

Similarly, the analysis of housekeeping circRNAs and their parental genes revealed several circular RNAs that are involved in key pathways related to fundamental cellular functions. For instance, the parental gene MAP2K1 of “circ1:164420445|164422619” encodes mitogen-activated protein kinase 1 (MAP2K1), which participates in the activation of the MAPK signaling pathway [47]. This pathway regulates critical biological processes such as cell growth, differentiation, proliferation, and survival. Another example is the parental gene ATG4C of “circ6:149633393|149666969”, which is involved in the regulation of autophagy, a highly regulated process essential for maintaining cellular survival and metabolic balance [48]. Autophagy plays a crucial role in cellular homeostasis by recycling damaged organelles and proteins. Additionally, the parental gene CREBBP of “circ3:38413987|38414699” and “circ3:38413987|38450234” is involved in the regulation of the cell cycle progression. CREBBP controls cell proliferation and differentiation fate, and it can interact with various apoptosis-regulating proteins, modulating the apoptotic signaling pathway in cells [49]. These findings indicate that housekeeping circRNAs derived from these parental genes are involved in important cellular pathways. These circRNAs likely contribute to the regulation of essential cellular functions, including cell growth, survival, metabolism, and differentiation. The identification of these housekeeping circRNAs and their associated parental genes sheds light on the functional roles of circRNAs in fundamental cellular processes. Understanding the regulatory mechanisms and functions of these housekeeping circRNAs can provide valuable insights into the molecular mechanisms underlying cellular homeostasis and cellular processes associated with development, differentiation, and disease.

Additionally, we investigated the binding capabilities of circRNAs to miRNAs based on their sequence complementarity. By evaluating the binding affinity between miRNAs and circRNAs, we identified a subset of circRNAs with strong binding capabilities, suggesting their potential role as competitive endogenous RNAs (ceRNAs). CeRNAs can sequester miRNAs and influence mRNA expression, forming a complex regulatory ceRNA network [19]. For example, in the liver, ebv-circLMP2A exists by regulating miR-3908, and miR-3908 further modulates the specifically expressed STAT2 [50,51].

Furthermore, we performed co-expression circRNA network analysis to examine the interactions among circRNAs. The analysis revealed that all circRNAs could be classified into 14 distinct modules, each representing a set of co-expressed circRNAs. Notably, nine of these modules exhibited strong correlations with individual tissues, suggesting their involvement in tissue-specific functions. From these modules, we identified hub circRNAs that likely play crucial roles in tissue-specific functions. Hub circRNAs are highly connected within the co-expression network and are considered key regulators within the ceRNA network. The identification of hub circRNAs provides valuable insights into the potential regulatory roles of circRNAs in tissue-specific processes.

Moreover, we conducted a conservation analysis to assess the conservation of circRNAs between humans and pigs. The results indicated that approximately 20% of the identified circRNAs share homologous genomic positions between the two species. The percentage is consistent with conservation studies between human and mouse [52]. This conservation suggests that these circRNAs may possess similar sequence and functional characteristics across species, highlighting their potential importance in cross-species biological processes.

Finally, although 24,745 circRNAs were identified in this study, the data volume needs further supplementation. There are a large number of genomic data in pigGTEx, but circRNA research is still lacking [53]. In the future, we hope to include more data, such as pigGTEx data, in the circRNA analysis, aiming to discover more novel circRNAs and thus more comprehensively complement the circRNA data.

Overall, our findings provide valuable insights into the circRNA expression profiles and their potential roles in the biological development and processes of various pig tissues. This information lays the foundation for further investigations into the functional significance and regulatory mechanisms of circRNAs in pigs, ultimately contributing to our understanding of tissue-specific gene regulation and the molecular basis of pig biology.

However, there are still some limitations in this study. Although Teng et al. have demonstrated that tissue-specific factors have a greater influence in transcriptome analysis than sex, breeding, age, and other factors [53], we categorized these factors as batch effects in our study due to the absence of some annotation information in the data. This approach, as compared to individually correcting for each factor, may introduce some deviation. Furthermore, the lack of corresponding phenotypic data means that we can only indirectly assess the relationship between circRNAs and phenotypes through association analysis between circRNAs and known trait-related QTLs.

In future research, we hope to have more comprehensive annotated data to obtain a more comprehensive pig circRNA profile, allowing for a more in-depth investigation into the relationship between circRNA expression and phenotypes.

## 4. Materials and Methods

### 4.1. Sample Collection

Firstly, in total, 215 samples were collected from the NCBI SRA database (https://www.ncbi.nlm.nih.gov/ accessed on 1 November 2022) across nine different pig tissues. The tissues included in this study were gallbladder (*n* = 16), heart (*n* = 6), liver (*n* = 25), longissimus dorsi (*n* = 22), lung (*n* = 23), ovary (*n* = 19), pituitary (*n* = 26), skeletal muscle (*n* = 46), and spleen (*n* = 32). Detailed sample information is shown in Table S1. The pig reference genome (*Sscrofa*11.1) was used for the analysis.

### 4.2. Quality Control and Alignment

The samples were transformed into pair-end fastq format using the fast-dump module in the sra-toolkit (version 2.8.2), and the quality of the raw sequencing data was assessed using FASTP software (version 0.23.2) with default parameters to filter and trim the reads to remove low-quality bases and adaptors [54]. Subsequently, the clean data were mapped to the reference genome using HISAT2 software (version 2.1.0), and the SAM files were converted to BAM format using Samtools (version v1.11) [55,56].

### 4.3. CircRNA Detection

To ensure the most reliable precision and sensitivity in circRNA identification, a combination of CIRI2 and find_cir methods were employed on the clean data [28,29]. In order to eliminate the impact of variables such as sex, breeding, age, and sequencing methods, we integrated them into batch effects and subsequently applied the combat algorithm from the R package sva after grouping the samples by their source for batch effect correction [57]. Subsequently, the number of circRNAs, their length, and the junction ratio (the proportion of circular junction reads compared to linear junction reads) were counted and quantified for each tissue. Additionally, three circRNA types were detected in each tissue, including exonic circRNAs originating from exons, intronic circRNAs originating from introns, and intergenetic circRNA originating from the intergenetic region.

### 4.4. CircRNA Profile between Tissues

To minimize potentially spurious events, only circRNAs detected in more than 50% of the samples within at least one tissue were considered for further analysis [58]. This filtering criterion resulted in a total of 24,745 circRNAs meeting the inclusion criteria. Subsequently, a t-distributed stochastic neighbor embedding (t-SNE) analysis was performed to explore the relationship and patterns among the samples [59], and based on the expression levels of circRNAs to assess the correlation between tissues, the Spearman’s correlation coefficient was calculated [60].

### 4.5. Identification of Housekeeping and Tissue-Specific CircRNAs

Two categories were defined to categorize circRNAs based on their expression patterns, housekeeping circRNAs, and tissue-specific circRNAs. The housekeeping circRNAs were identified by the conditions for the housekeeping mRNA identification, as in a previous study [61]. On the other hand, tissue-specific circRNAs were defined as those expressed exclusively in one tissue. To visualize the expression patterns of circRNAs across tissues, a heatmap was generated using the Pheatmap package of R (version 1.0.12).

### 4.6. GO and KEGG Enrichment

Two tools were utilized to analyze the parental genes of the identified circRNAs and gain insights into their potential functional roles, the clusterProfiler package of R for Gene Ontology (GO) enrichment analysis and the ShinyGO online server (http://bioinformatics.sdstate.edu/go/ accessed on 1 January 2023) for Kyoto Encyclopedia of Genes and Genomes (KEGG) pathway enrichment analysis [62,63]. In both analyses, the hypergeometric test was applied to determine the significance of enrichment. A significance threshold of *p* < 0.05 was considered, indicating that genes associated with circRNAs were significantly enriched in specific GO terms or KEGG pathways.

### 4.7. Competing Endogenous RNA Network Construction

To investigate the potential regulatory interactions between circRNAs (housekeeping circRNAs and tissue-specific circRNAs), miRNAs, and mRNAs, miRanda software (version 3.3a) was used to predict the interaction between circRNA–miRNA and miRNA–mRNA, a match score above 140 was used as a threshold to predict significant interactions [64]. Firstly, the miRNA sequences were obtained from the miRBase database (http://www.mirbase.org/ accessed on 1 March 2023) [65], and the sequences of circRNAs and mRNAs were obtained from a previous study using bedtools2 software (version 2.23.0) [66,67]. Subsequently, miRanda was used to calculate the free energy and score between miRNAs and circRNAs, as well as between miRNAs and mRNAs. These calculations provided information about the stability and potential strength of interactions between miRNAs and their targets. Finally, the regulatory interactions among circRNA–miRNA–mRNA were visualized using Cytoscape software (version 3.10.0) [68].

### 4.8. Co-Expression Network Analysis

The Weighted Gene Co-expression Network Analysis (WGCNA) method was employed to investigate the co-expression patterns of circRNAs among tissues [69]. The hclust function with the average agglomeration method for the WGCNA package was used for cluster analysis. The pickSoftThreshold function from the WGCNA package was utilized to determine the soft threshold (*β*), which determines the connectivity strength between circRNAs. The module with a correlation coefficient larger than 0.65 was considered the important tissue-specific module in this study [61]. Subsequently, the important tissue-specific modules were identified from the co-expression network and further analyzed to identify core circRNAs within them. Briefly, the key circRNAs within the tissue-specific modules, which are called hub circRNAs, were identified using Module Membership (MM) and Gene Significance (GS) methods. Hub circRNAs were determined based on predefined thresholds, typically |GS| > 0.2 and |MM| > 0.8, indicating strong correlations between circRNAs and module eigengenes [70].

### 4.9. Association Analysis of Traits

To identify the relationship between tissue-specific circRNAs and phenotypes, the QTL data of pigs were downloaded from the QTLdb database (https://www.animalgenome.org/cgi-bin/QTLdb/index accessed on 1 November 2023), and we counted any QTLs that overlapped with circRNAs in terms of location and subsequently performed Fisher’s tests to obtain the degree of association between circRNA and trait-related QTL [71].

### 4.10. Conservation Analysis

To identify and compare circRNAs between pig and human, the predicted circRNAs were converted from pig genomic locations (susScr11) to human genomic locations (hg19) by using the LiftOver tool from the UCSC genome browser with the parameter “minMatch = 0.5” [72]. The circRNAs aligned to the human genome were then compared with the known circRNAs in humans, which were obtained from the circBase database (http://www.circbase.org/ accessed on 1 May 2023) [31].

## 5. Conclusions

In summary, our study focused on exploring the expression profiles of circRNAs in nine tissues of pigs. We successfully identified 492 housekeeping circRNAs and 3489 tissue-specific circRNAs, highlighting their distinct expression patterns and potential roles in tissue-specific processes. Moreover, we obtained 14 modules consisting of 1210 hub circRNAs which are likely to play crucial regulatory roles in various biological activities across tissues. Notably, we have discovered a total of 3818 novel circRNAs, contributing additional information to augment the pig circRNA dataset.

## Figures and Tables

**Table 1:** Number of conservation circRNAs between pig and human.

	Number	Frequency
Detected CircRNAs	24,745	/
Aligned CircRNAs	19,078	77.10%
Conserved CircRNAs	4774	19.29%

## Data Availability

The data presented in this study are available in this article (Table S1).

## Supplementary Materials

The supporting information can be downloaded at: https://www.mdpi.com/article/10.3390/ijms242216205/s1.

## References

[1] Sanger H.L., Klotz G., Riesner D., Kleinschmidt G. (1976). Viroids are single-strandedcovalently closed circular RNA molecules existing as highly base-paired rod-like structures. Proc. Natl. Acad. Sci. USA.

[2] Lei M., Zheng G., Ning Q., Zheng J., Dong D. (2020). Translation and functional roles of circular RNAs in human cancer. Mol. Cancer.

[3] Wang Y., Zhang Y., Wang P., Fu X., Lin W. (2020). Circular RNAs in renal cell carcinoma: Implications for tumorigenesis, diagnosis, and therapy. Mol. Cancer.

[4] Salzman J., Gawad C., Wang P.L., Lacayo N., Brown P.O. (2012). Circular RNAs are the predominant transcript isoform from hundreds of human genes in diverse cell types. PLoS ONE.

[5] Du W.W., Yang W., Liu E., Yang Z., Dhaliwal P., Yang B.B. (2016). Foxo3 circular RNA retards cell cycle progression via forming ternary complexes with p21 and CDK2. Nucleic Acids Res..

[6] Holdt L.M., Kohlmaier A., Teupser D. (2018). Molecular roles and function of circular RNAs in eukaryotic cells. Cell. Mol. Life Sci..

[7] Li Z., Huang C., Bao C., Chen L., Lin M., Wang X., Zhong G., Yu B., Hu W., Dai L. (2015). Exon-intron circular RNAs regulate transcription in the nucleus. Nat. Struct. Mol. Biol..

[8] Kristensen L.S., Andersen M.S., Stagsted L.V.W., Ebbesen K.K., Hansen T.B., Kjems J. (2019). The biogenesis, biology and characterization of circular RNAs. Nat. Rev. Genet..

[9] Zhang Y., Zhang X.O., Chen T., Xiang J.F., Yin Q.F., Xing Y.H., Zhu S., Yang L., Chen L.L. (2013). Circular intronic long noncoding RNAs. Mol. Cell.

[10] Xiao M.S., Ai Y., Wilusz J.E. (2020). Biogenesis and Functions of Circular RNAs Come into Focus. Trends Cell Biol..

[11] Liu L., Gu M., Ma J., Wang Y., Li M., Wang H., Yin X., Li X. (2022). CircGPR137B/miR-4739/FTO feedback loop suppresses tumorigenesis and metastasis of hepatocellular carcinoma. Mol. Cancer.

[12] Li Z., Yang H.Y., Dai X.Y., Zhang X., Huang Y.Z., Shi L., Wei J.F., Ding Q. (2021). CircMETTL3, upregulated in a m6A-dependent manner, promotes breast cancer progression. Int. J. Biol. Sci..

[13] Abdelmohsen K., Panda A.C., Munk R., Grammatikakis I., Dudekula D.B., De S., Kim J., Noh J.H., Kim K.M., Martindale J.L. (2017). Identification of HuR target circular RNAs uncovers suppression of PABPN1 translation by CircPABPN1. RNA Biol..

[14] Alberio R. (2020). Regulation of Cell Fate Decisions in Early Mammalian Embryos. Annu. Rev. Anim. Biosci..

[15] Chen K., Baxter T., Muir W.M., Groenen M.A., Schook L.B. (2007). Genetic resources, genome mapping and evolutionary genomics of the pig (Sus scrofa). Int. J. Biol. Sci..

[16] Li M., Tian S., Jin L., Zhou G., Li Y., Zhang Y., Wang T., Yeung C.K., Chen L., Ma J. (2013). Genomic analyses identify distinct patterns of selection in domesticated pigs and Tibetan wild boars. Nat. Genet..

[17] Li M., Zhang N., Li J., Ji M., Zhao T., An J., Cai C., Yang Y., Gao P., Cao G. (2023). CircRNA Profiling of Skeletal Muscle in Two Pig Breeds Reveals CircIGF1R Regulates Myoblast Differentiation via miR-16. Int. J. Mol. Sci..

[18] Wang J., Chen J.F., Ma Q., Mo D.L., Sun J.J., Ren Q.L., Zhang J.Q., Lu Q.X., Xing B.S. (2022). Identification and characterization of circRNAs related to meat quality during embryonic development of the longissimus dorsi muscle in two pig breeds. Front. Genet..

[19] Zhuang X., Lin Z., Xie F., Luo J., Chen T., Xi Q., Zhang Y., Sun J. (2022). Identification of circRNA-associated ceRNA networks using longissimus thoracis of pigs of different breeds and growth stages. BMC Genom..

[20] Niu X., Huang Y., Lu H., Li S., Huang S., Ran X., Wang J. (2022). CircRNAs in Xiang pig ovaries among diestrus and estrus stages. Porc. Health Manag..

[21] Mester-Tonczar J., Winkler J., Einzinger P., Hasimbegovic E., Kastner N., Lukovic D., Zlabinger K., Spannbauer A., Traxler D., Batkai S. (2020). Association between Circular RNA CDR1as and Post-Infarction Cardiac Function in Pig Ischemic Heart Failure: Influence of the Anti-Fibrotic Natural Compounds Bufalin and Lycorine. Biomolecules.

[22] Groenen M.A. (2016). A decade of pig genome sequencing: A window on pig domestication and evolution. Genet. Sel. Evol..

[23] Lv W., Jin J., Xu Z., Guo Y., Wang X., Wang S., Zhang J., Zuo H., Bai W., Peng Y. (2020). lncMGPF is a novel positive regulator of muscle growth and regeneration. J. Cachexia Sarcopenia Muscle.

[24] You X., Liu M., Liu Q., Li H., Qu Y., Gao X., Huang C., Luo G., Cao G., Xu D. (2022). miRNA let-7 family regulated by NEAT1 and ARID3A/NF-κB inhibits PRRSV-2 replication in vitro and in vivo. PLoS Pathog..

[25] Meng X., Hu D., Zhang P., Chen Q., Chen M. (2019). CircFunBase: A database for functional circular RNAs. Database.

[26] Wu W., Ji P., Zhao F. (2020). CircAtlas: An integrated resource of one million highly accurate circular RNAs from 1070 vertebrate transcriptomes. Genome Biol..

[27] Jin L., Tang Q., Hu S., Chen Z., Zhou X., Zeng B., Wang Y., He M., Li Y., Gui L. (2021). A pig BodyMap transcriptome reveals diverse tissue physiologies and evolutionary dynamics of transcription. Nat. Commun..

[28] Gao Y., Wang J., Zhao F. (2015). CIRI: An efficient and unbiased algorithm for de novo circular RNA identification. Genome Biol..

[29] Hansen T.B., Venø M.T., Damgaard C.K., Kjems J. (2016). Comparison of circular RNA prediction tools. Nucleic Acids Res..

[30] Pan X., Gong W., He Y., Li N., Zhang H., Zhang Z., Li J., Yuan X. (2021). Ovary-derived circular RNAs profile analysis during the onset of puberty in gilts. BMC Genomics.

[31] Glažar P., Papavasileiou P., Rajewsky N. (2014). circBase: A database for circular RNAs. RNA.

[32] Tian J., Fu Y., Li Q., Xu Y., Xi X., Zheng Y., Yu L., Wang Z., Yu B., Tian J. (2020). Differential Expression and Bioinformatics Analysis of CircRNA in PDGF-BB-Induced Vascular Smooth Muscle Cells. Front. Genet..

[33] Zhang Y., Guo X., Pei J., Chu M., Ding X., Wu X., Liang C., Yan P. (2020). CircRNA Expression Profile during Yak Adipocyte Differentiation and Screen Potential circRNAs for Adipocyte Differentiation. Genes.

[34] Ruan H., Xiang Y., Ko J., Li S., Jing Y., Zhu X., Ye Y., Zhang Z., Mills T., Feng J. (2019). Comprehensive characterization of circular RNAs in ~ 1000 human cancer cell lines. Genome Med..

[35] Rybak-Wolf A., Stottmeister C., Glažar P., Jens M., Pino N., Giusti S., Hanan M., Behm M., Bartok O., Ashwal-Fluss R. (2015). Circular RNAs in the Mammalian Brain Are Highly Abundant, Conserved, and Dynamically Expressed. Mol. Cell.

[36] Pérez-Hernández M., van Opbergen C.J.M., Bagwan N., Vissing C.R., Marrón-Liñares G.M., Zhang M., Torres Vega E., Sorrentino A., Drici L., Sulek K. (2022). Loss of Nuclear Envelope Integrity and Increased Oxidant Production Cause DNA Damage in Adult Hearts Deficient in PKP2: A Molecular Substrate of ARVC. Circulation.

[37] Cherian A.V., Fukuda R., Augustine S.M., Maischein H.M., Stainier D.Y. (2016). N-cadherin relocalization during cardiac trabeculation. Proc. Natl. Acad. Sci. USA.

[38] Zhang X., Yin H., Zhang X., Jiang X., Liu Y., Zhang H., Peng Y., Li D., Yu Y., Zhang J. (2022). N6-methyladenosine modification governs liver glycogenesis by stabilizing the glycogen synthase 2 mRNA. Nat. Commun..

[39] Schartner V., Romero N.B., Donkervoort S., Treves S., Munot P., Pierson T.M., Dabaj I., Malfatti E., Zaharieva I.T., Zorzato F. (2017). Dihydropyridine receptor (DHPR, CACNA1S) congenital myopathy. Acta Neuropathol..

[40] McKelvey M.C., Abladey A.A., Small D.M., Doherty D.F., Williams R., Scott A., Spek C.A., Borensztajn K.S., Holsinger L., Booth R. (2022). Cathepsin S Contributes to Lung Inflammation in Acute Respiratory Distress Syndrome. Am. J. Respir. Crit. Care Med..

[41] Zhou Y., Fu B., Xu X., Zhang J., Tong X., Wang Y., Dong Z., Zhang X., Shen N., Zhai Y. (2020). PBX1 expression in uterine natural killer cells drives fetal growth. Sci. Transl. Med..

[42] Qi C., Sorrentino S., Medalia O., Korkhov V.M. (2019). The structure of a membrane adenylyl cyclase bound to an activated stimulatory G protein. Science.

[43] Yang N., MacArthur D.G., Gulbin J.P., Hahn A.G., Beggs A.H., Easteal S., North K. (2003). ACTN3 genotype is associated with human elite athletic performance. Am. J. Hum. Genet..

[44] Glawe J.D., Patrick D.R., Huang M., Sharp C.D., Barlow S.C., Kevil C.G. (2009). Genetic deficiency of Itgb2 or ItgaL prevents autoimmune diabetes through distinctly different mechanisms in NOD/LtJ mice. Diabetes.

[45] Brown M.R., Holmes H., Rakshit K., Javeed N., Her T.K., Stiller A.A., Sen S., Shull G.E., Prakash Y.S., Romero M.F. (2021). Electrogenic sodium bicarbonate cotransporter NBCe1 regulates pancreatic β cell function in type 2 diabetes. J. Clin. Investig..

[46] Andersson E.R., Chivukula I.V., Hankeova S., Sjöqvist M., Tsoi Y.L., Ramsköld D., Masek J., Elmansuri A., Hoogendoorn A., Vazquez E. (2018). Mouse Model of Alagille Syndrome and Mechanisms of Jagged1 Missense Mutations. Gastroenterology.

[47] Fang J.Y., Richardson B.C. (2005). The MAPK signalling pathways and colorectal cancer. Lancet Oncol..

[48] Devis-Jauregui L., Eritja N., Davis M.L., Matias-Guiu X., Llobet-Navàs D. (2021). Autophagy in the physiological endometrium and cancer. Autophagy.

[49] Huang Y.H., Cai K., Xu P.P., Wang L., Huang C.X., Fang Y., Cheng S., Sun X.J., Liu F., Huang J.Y. (2021). CREBBP/EP300 mutations promoted tumor progression in diffuse large B-cell lymphoma through altering tumor-associated macrophage polarization via FBXW7-NOTCH-CCL2/CSF1 axis. Signal Transduct. Target. Ther..

[50] Liu X., Chen J., Zhang J. (2017). AdipoR1-mediated miR-3908 inhibits glioblastoma tumorigenicity through downregulation of STAT2 associated with the AMPK/SIRT1 pathway. Oncol. Rep..

[51] Gong L.P., Chen J.N., Dong M., Xiao Z.D., Feng Z.Y., Pan Y.H., Zhang Y., Du Y., Zhang J.Y., Bi Y.H. (2020). Epstein-Barr virus-derived circular RNA LMP2A induces stemness in EBV-associated gastric cancer. EMBO Rep..

[52] Guo J.U., Agarwal V., Guo H., Bartel D.P. (2014). Expanded identification and characterization of mammalian circular RNAs. Genome Biol..

[53] Teng J., Gao Y., Yin H., Bai Z., Liu S., Zeng H., Bai L., Cai Z., Zhao B., Li X. (2022). A compendium of genetic regulatory effects across pig tissues. bioRxiv.

[54] Chen S., Zhou Y., Chen Y., Gu J. (2018). fastp: An ultra-fast all-in-one FASTQ preprocessor. Bioinformatics.

[55] Kim D., Langmead B., Salzberg S.L. (2015). HISAT: A fast spliced aligner with low memory requirements. Nat. Methods.

[56] Li H., Handsaker B., Wysoker A., Fennell T., Ruan J., Homer N., Marth G., Abecasis G., Durbin R., 1000 Genome Project Data Processing Subgroup (2009). The Sequence Alignment/Map format and SAMtools. Bioinformatics.

[57] Zhang Y., Parmigiani G., Johnson W.E. (2020). ComBat-seq: Batch effect adjustment for RNA-seq count data. NAR Genom. Bioinform..

[58] Chen Y.J., Chen C.Y., Mai T.L., Chuang C.F., Chen Y.C., Gupta S.K., Yen L., Wang Y.D., Chuang T.J. (2020). Genome-wide, integrative analysis of circular RNA dysregulation and the corresponding circular RNA-microRNA-mRNA regulatory axes in autism. Genome Res..

[59] Kobak D., Berens P. (2019). The art of using t-SNE for single-cell transcriptomics. Nat. Commun..

[60] Spearman C. (2010). The proof and measurement of association between two things. Int. J. Epidemiol..

[61] Zhang T., Wang T., Niu Q., Xu L., Chen Y., Gao X., Gao H., Zhang L., Liu G.E., Li J. (2022). Transcriptional atlas analysis from multiple tissues reveals the expression specificity patterns in beef cattle. BMC Biol..

[62] Yu G., Wang L.G., Yan G.R., He Q.Y. (2015). DOSE: An R/Bioconductor package for disease ontology semantic and enrichment analysis. Bioinformatics.

[63] Ge S.X., Jung D., Yao R. (2020). ShinyGO: A graphical gene-set enrichment tool for animals and plants. Bioinformatics.

[64] Betel D., Koppal A., Agius P., Sander C., Leslie C. (2010). Comprehensive modeling of microRNA targets predicts functional non-conserved and non-canonical sites. Genome Biol..

[65] Kozomara A., Birgaoanu M., Griffiths-Jones S. (2019). miRBase: From microRNA sequences to function. Nucleic Acids Res..

[66] Pan X., Cai J., Wang Y., Xu D., Jiang Y., Gong W., Tian Y., Shen Q., Zhang Z., Yuan X. (2022). Expression Profile of Housekeeping Genes and Tissue-Specific Genes in Multiple Tissues of Pigs. Animals.

[67] Quinlan A.R. (2014). BEDTools: The Swiss-Army Tool for Genome Feature Analysis. Curr. Protoc. Bioinformatics.

[68] Shannon P., Markiel A., Ozier O., Baliga N.S., Wang J.T., Ramage D., Amin N., Schwikowski B., Ideker T. (2003). Cytoscape: A software environment for integrated models of biomolecular interaction networks. Genome Res..

[69] Langfelder P., Horvath S. (2008). WGCNA: An R package for weighted correlation network analysis. BMC Bioinform..

[70] Liang W., Sun F., Zhao Y., Shan L., Lou H. (2020). Identification of Susceptibility Modules and Genes for Cardiovascular Disease in Diabetic Patients Using WGCNA Analysis. J. Diabetes Res..

[71] Hu Z.L., Park C.A., Reecy J.M. (2022). Bringing the Animal QTLdb and CorrDB into the future: Meeting new challenges and providing updated services. Nucleic Acids Res..

[72] Kent W.J., Sugnet C.W., Furey T.S., Roskin K.M., Pringle T.H., Zahler A.M., Haussler D. (2002). The human genome browser at UCSC. Genome Res..

