# Supplementary material for: Comprehensive Analysis of CircRNA Expression Profiles in Multiple Tissues of Pigs
Source: Int J Mol Sci. 2023 Nov 11;24(22):16205. doi: 10.3390/ijms242216205 (PMC10671760; doi:10.3390/ijms242216205)
Supplement: Supplementary file 1 [file ijms-24-16205-s001.zip › Supplementary Figure.pdf]

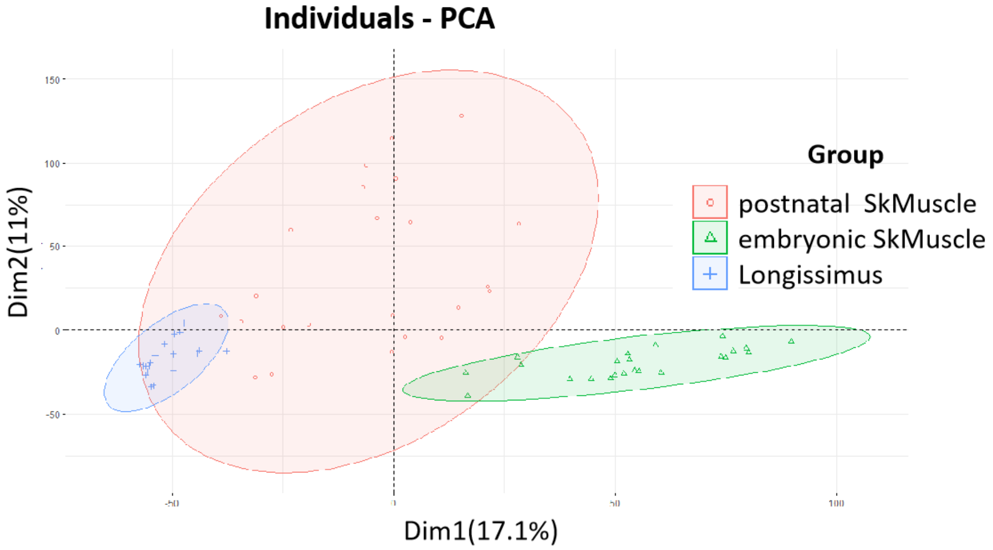

Figure S1. The PCA analyse among postnatal skeletal muscle, embryoic skeletal muscle and longissimus dorsi.

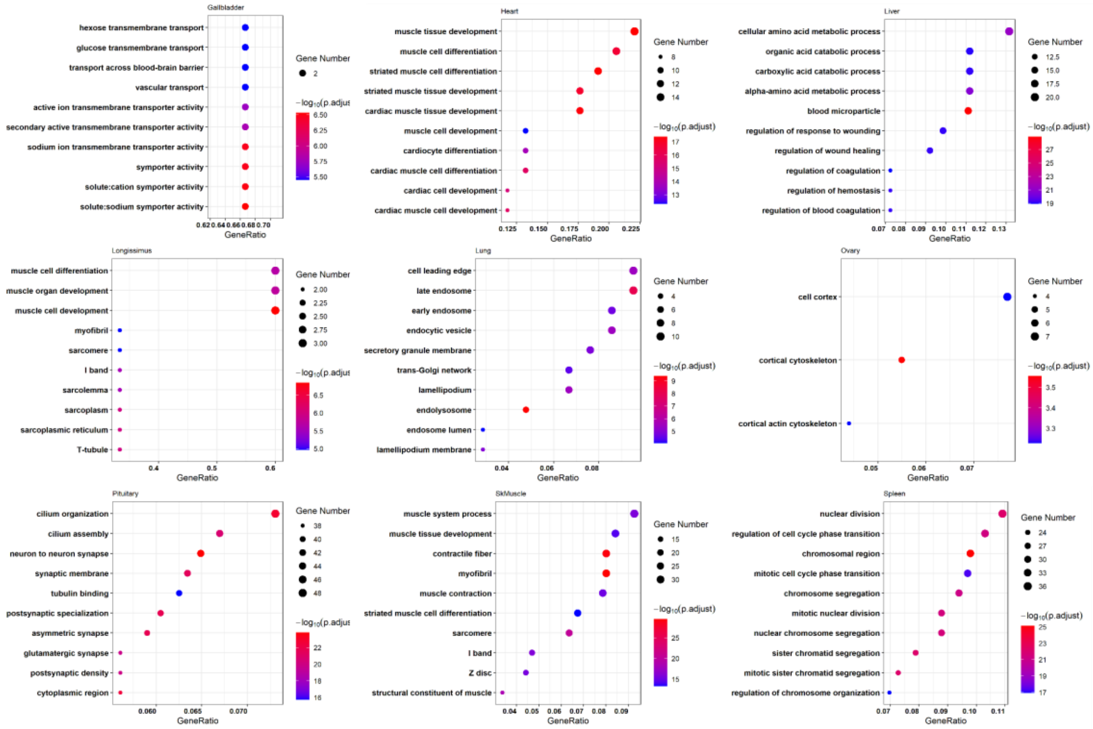

Figure S2. The GO analyse of tissue-specific circRNA's parental genes.

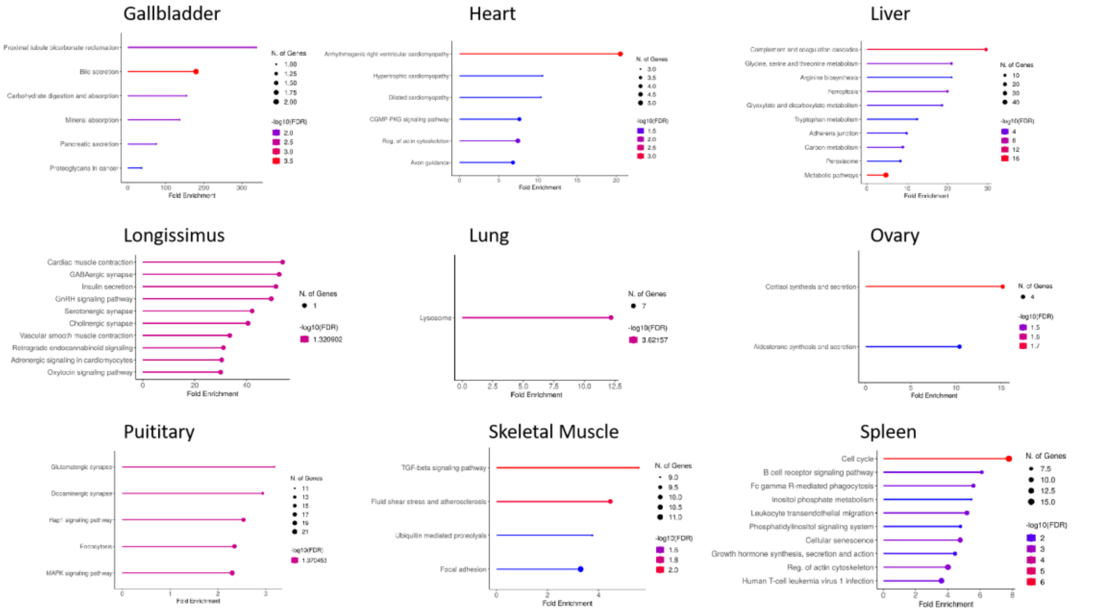

Figure S3. The KEGG analyse of tissue-specific circRNA's parental genes.

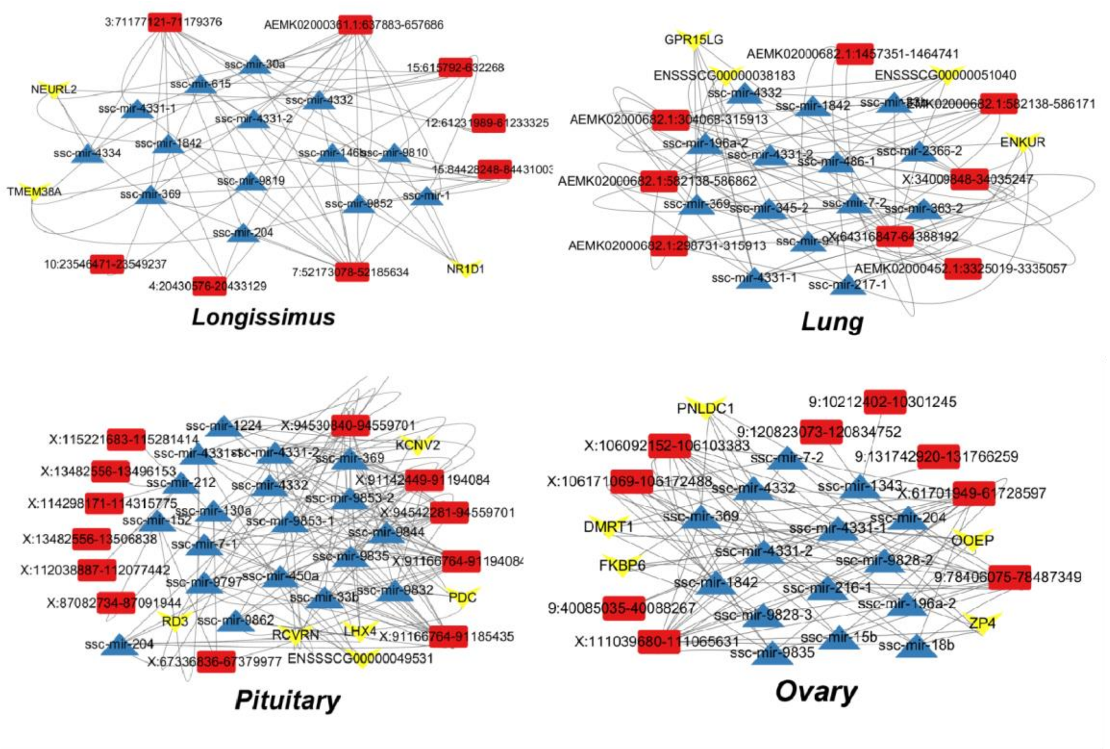

Figure S4. The ceRNA network between tissue-specific circRNAs and tissue-specific genes in longissimus dorsi, lung, pituitary and ovary.

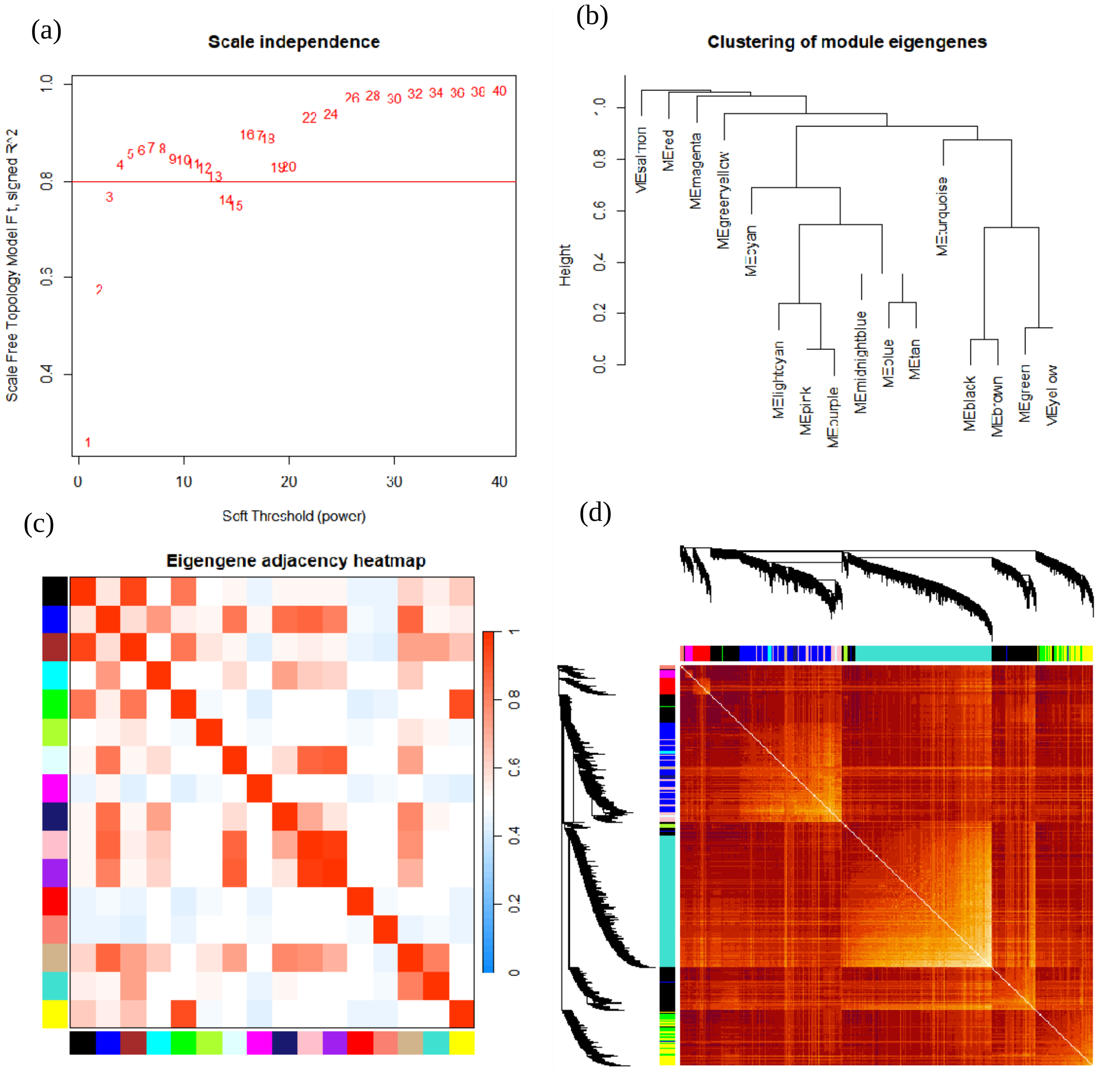

Figure S5. The weighted gene co-expression network analyse of circRNAs. (a) The determination of the soft threshold (β). (b) The clustering relationship among modules. (c) The correlation among modules. (d) The correlation among circRNAs.

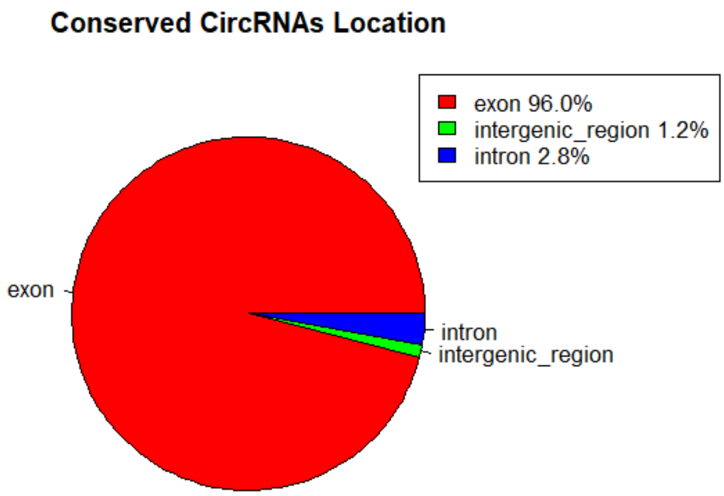

Figure S6. The location of conserved circRNAs between human and pigs
